# Supplementary material for: ERK3 Increases Snail Protein Stability by Inhibiting FBXO11-Mediated Snail Ubiquitination
Source: Cancers (Basel). 2023 Dec 24;16(1):105. doi: 10.3390/cancers16010105 (PMC10777929; doi:10.3390/cancers16010105)

Figure S1

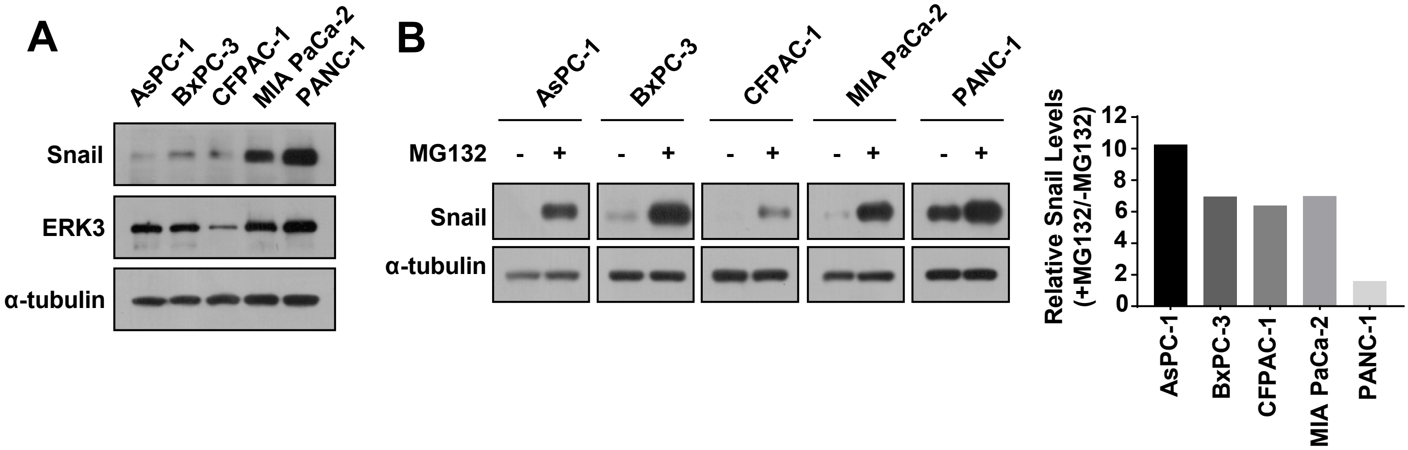

Figure S1. Endogenous Snail protein levels in pancreatic cancer cell lines. (**A**) Immunoblot analysis in pancreatic cancer cell lines with specific antibody against Snail or ERK3. (**B**) Pancreatic cancer cell lines treated with 10μM MG132 for 12hr. Cell lysates were immunoblotted with antibody against Snail.

Figure S2

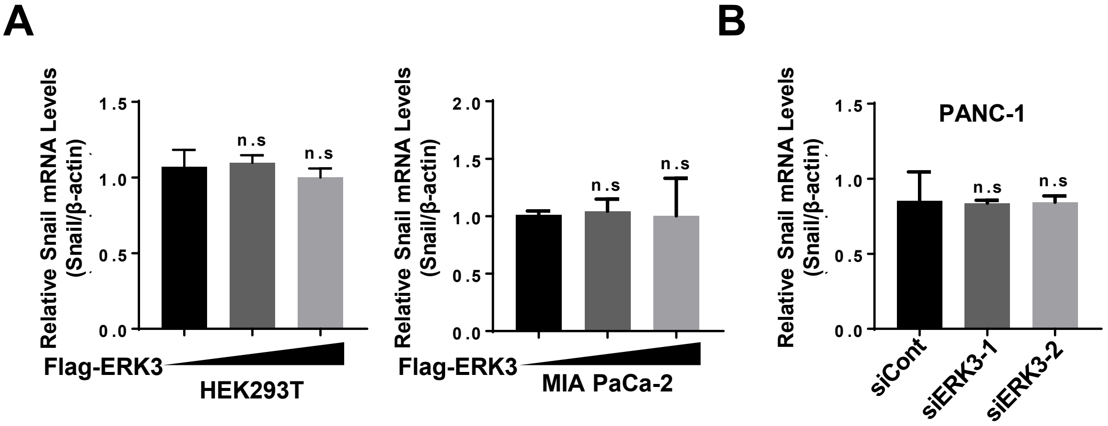

Figure S2. mRNA levels of Snail Endogenous Snail in ERK3 expressing or ERK3-depleted pancreatic cancer cells. (**A**) ERK3 was transfected into HEK293T or MIA PaCa-2 cells. mRNA was extracted from each cell lysate and mRNA level of Snail was analyzed by qRT-PCR. (**B**) ERK3-specific siRNAs were transfected into PANC-1 cells. mRNA was extracted from each cell lysate and mRNA level of Snail was analyzed by qRT-PCR.

Figure S3

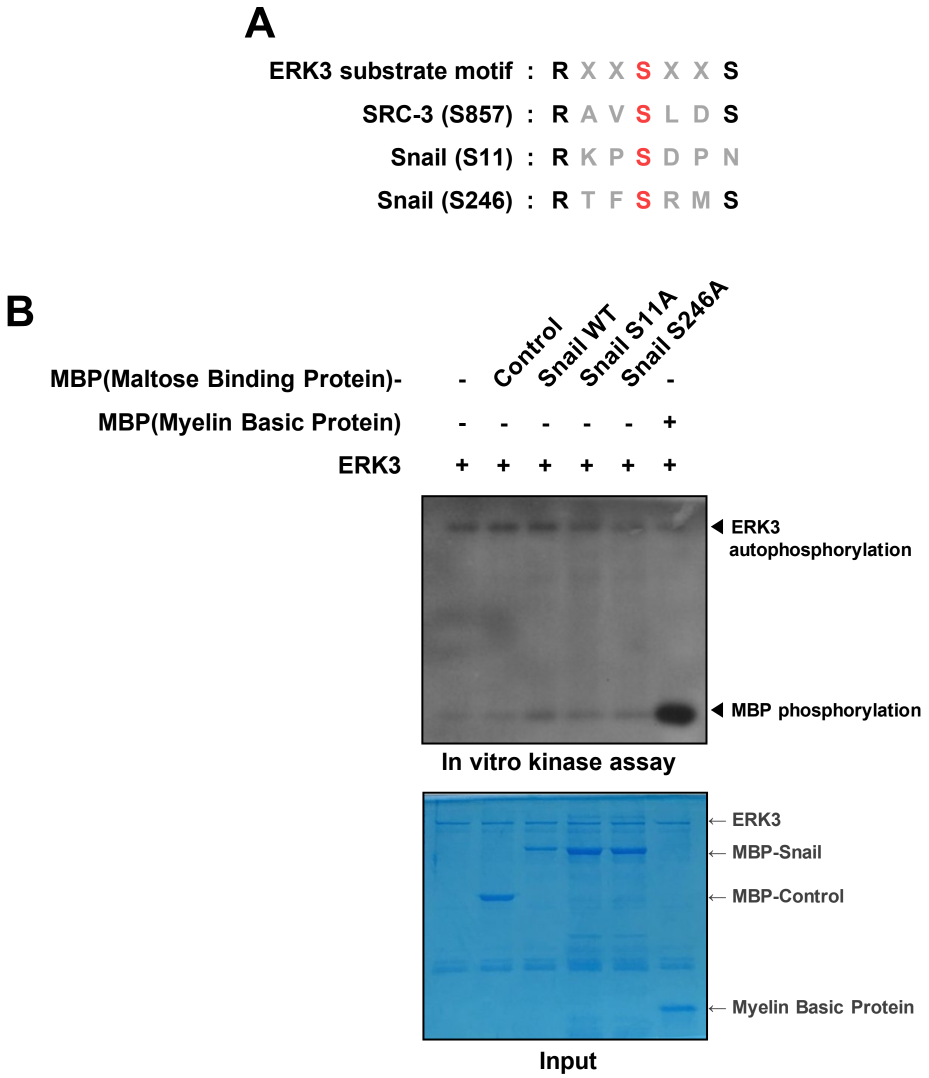

Figure S3. ERK3 cannot phosphorylate Snail in vitro. (**A**) ERK3 substrate motif in Snail protein sequence. Red seine residues are phosphorylation site. (**B**) In vitto kinase assays were performed by incubating purified recombinant active ERK3 protein with purified MBP-fused wild-type (WT)-Snail or serine mutants (S11A or S246A) in the presence of [γ32P]-ATP. The resultant products were subjected to SDS polyacrylamide gel electrophoresis and autoradiography.

Figure S4

Figure 1A

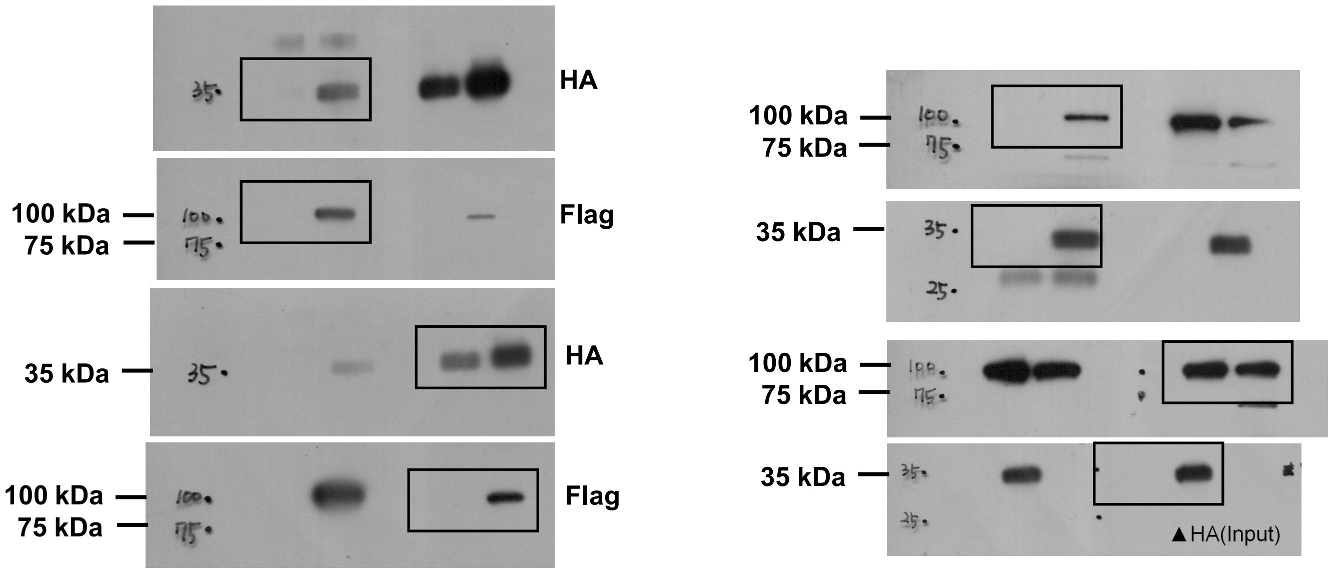

Figure 1B

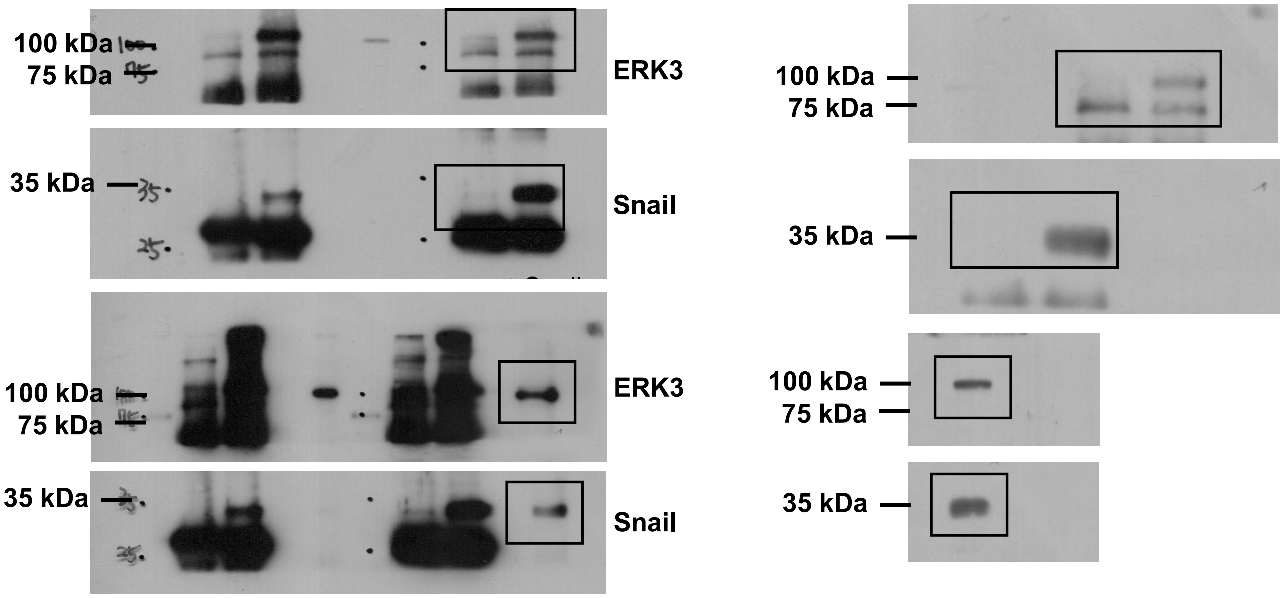

Figure S5

Figure 1C

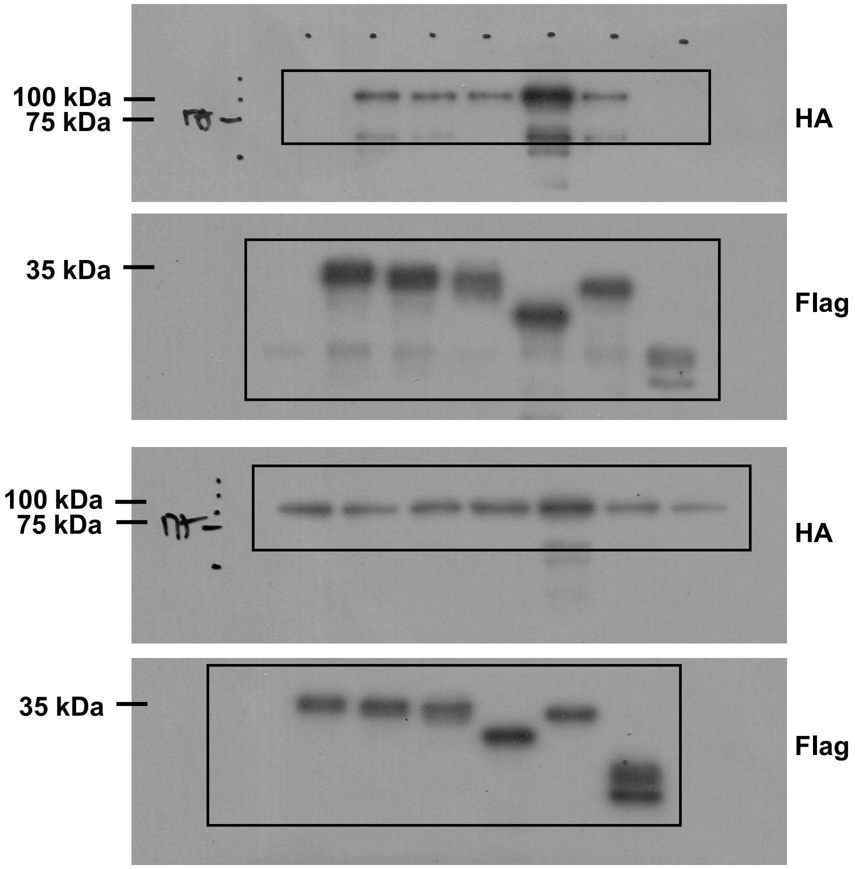

Figure S6

Figure 4A

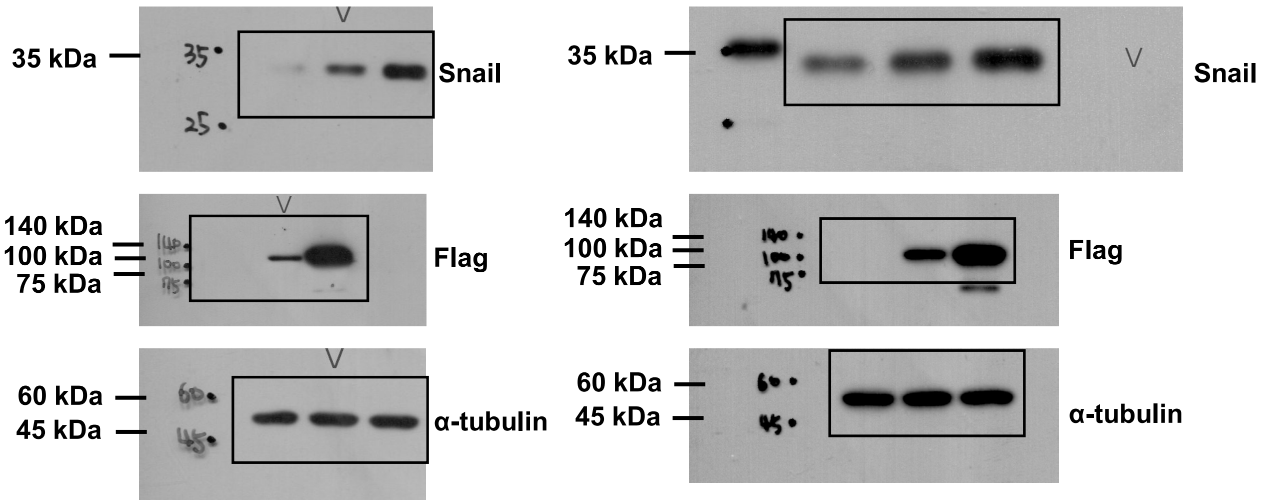

Figure 4B

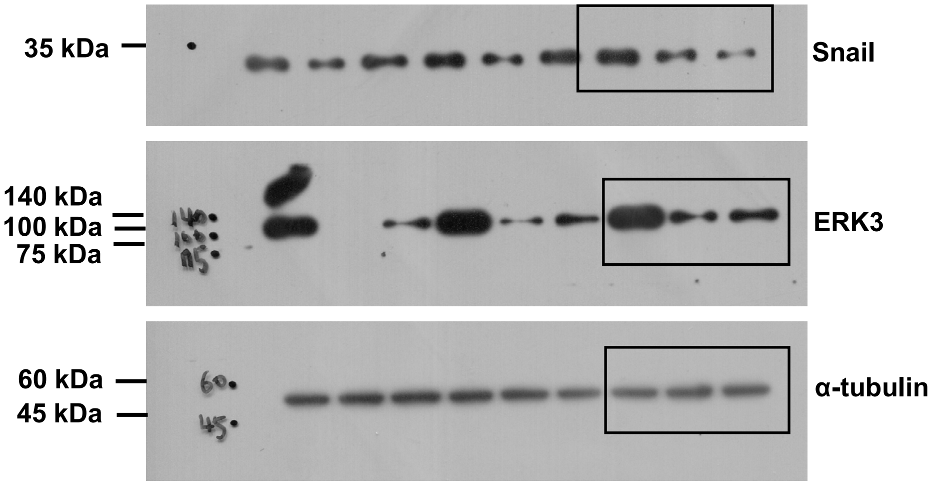

Figure S7

Figure 4C

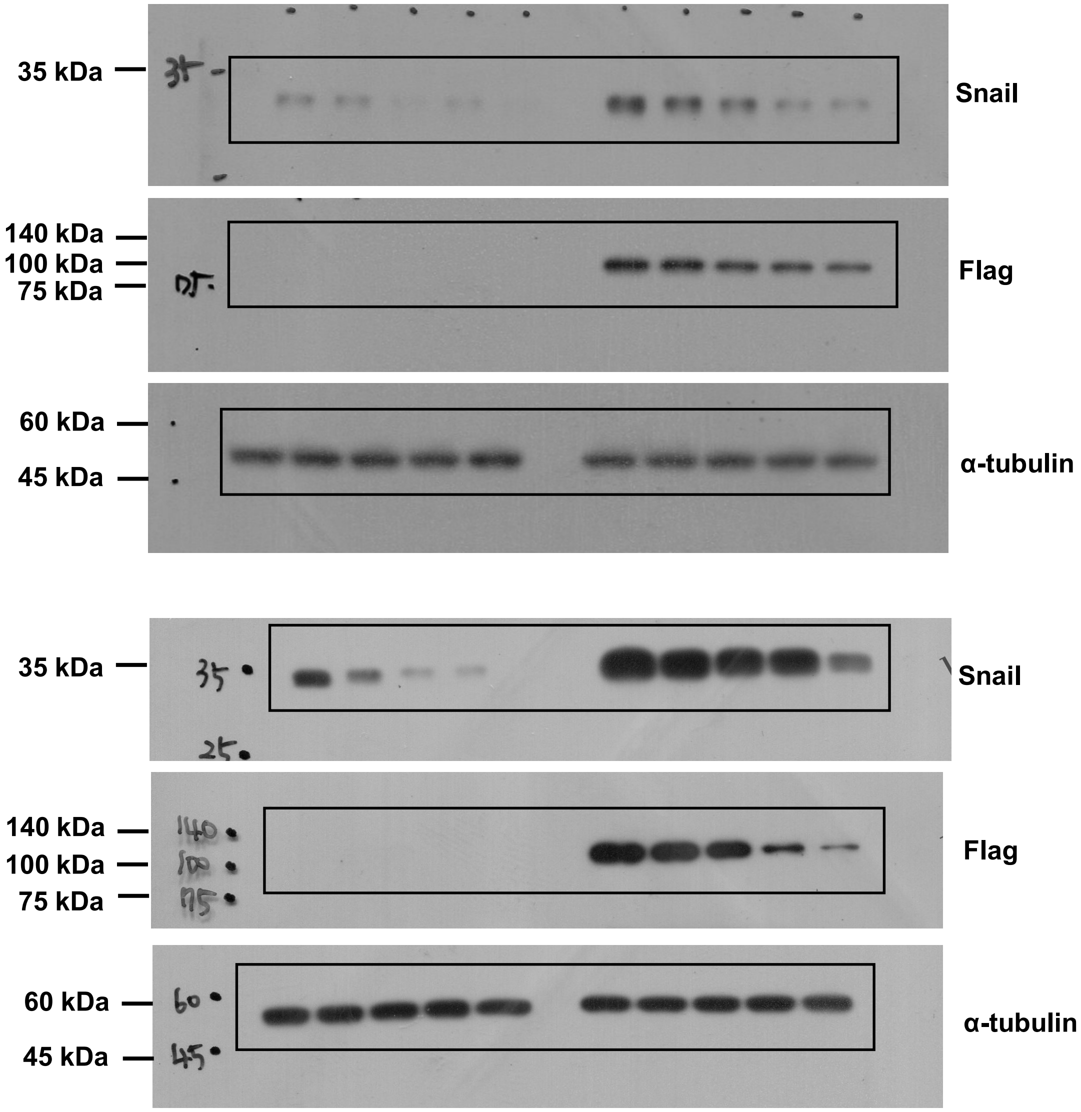

Figure 4D

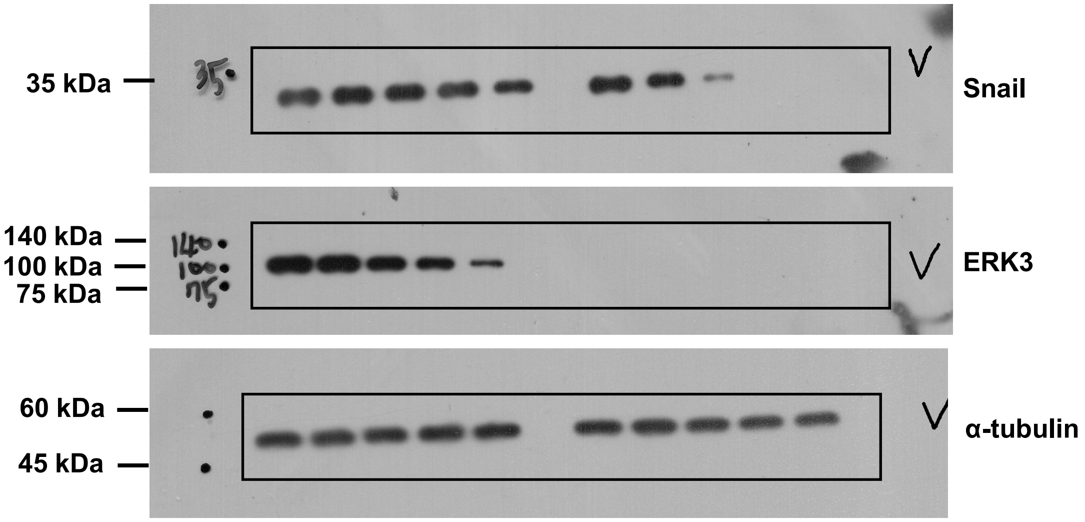

Figure S8

Figure 4E

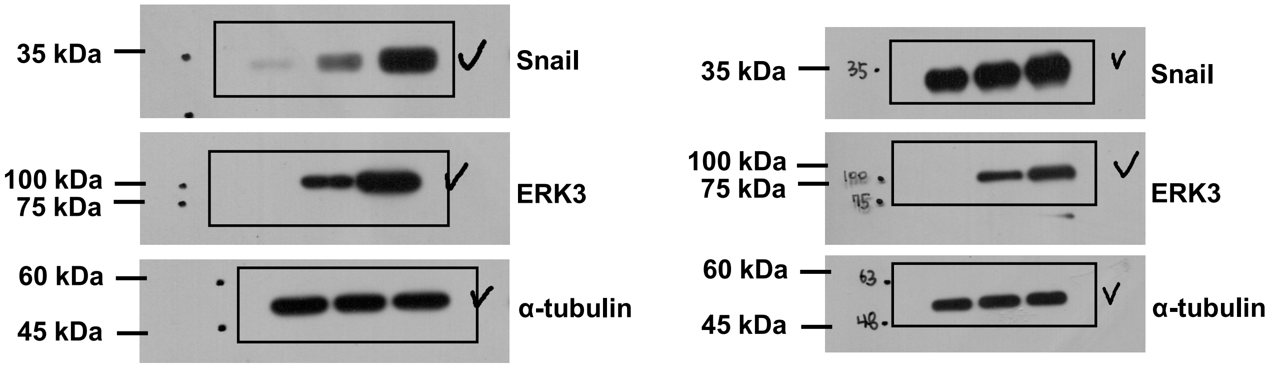

Figure 4F

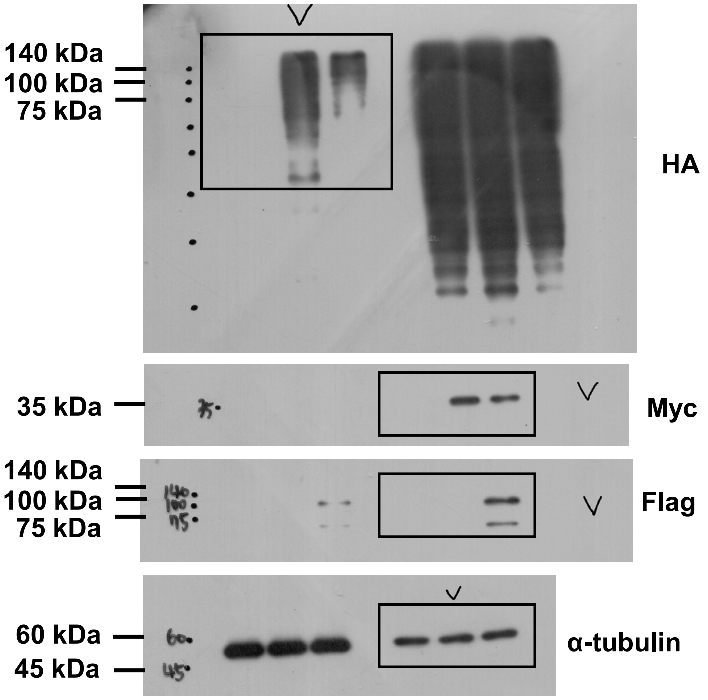

Figure 4H

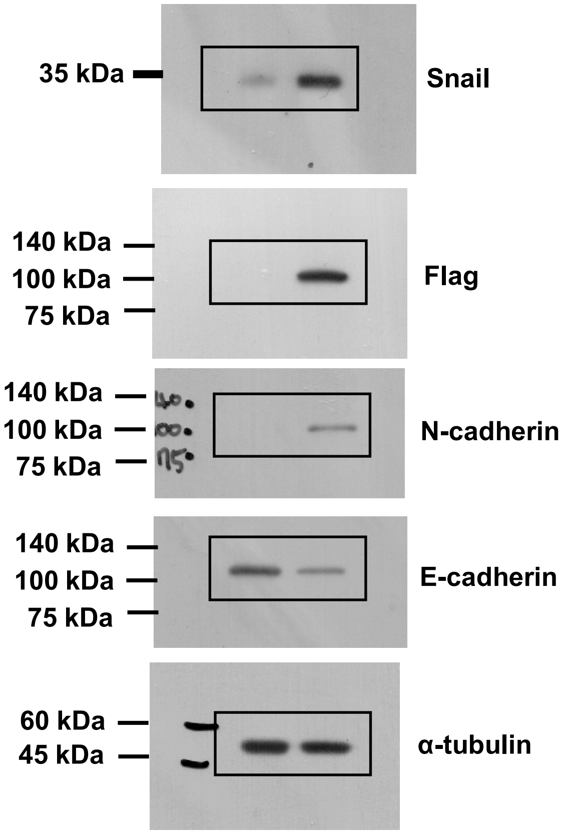

Figure S9

Figure 5A

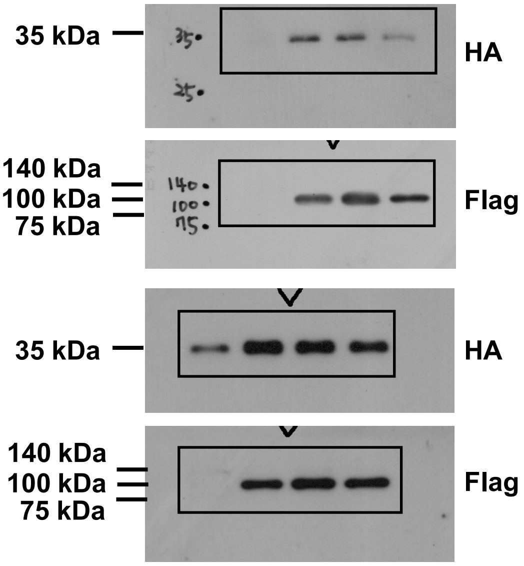

Figure 5B

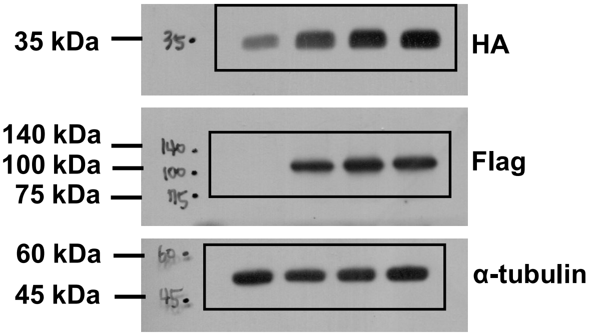

Figure 5C

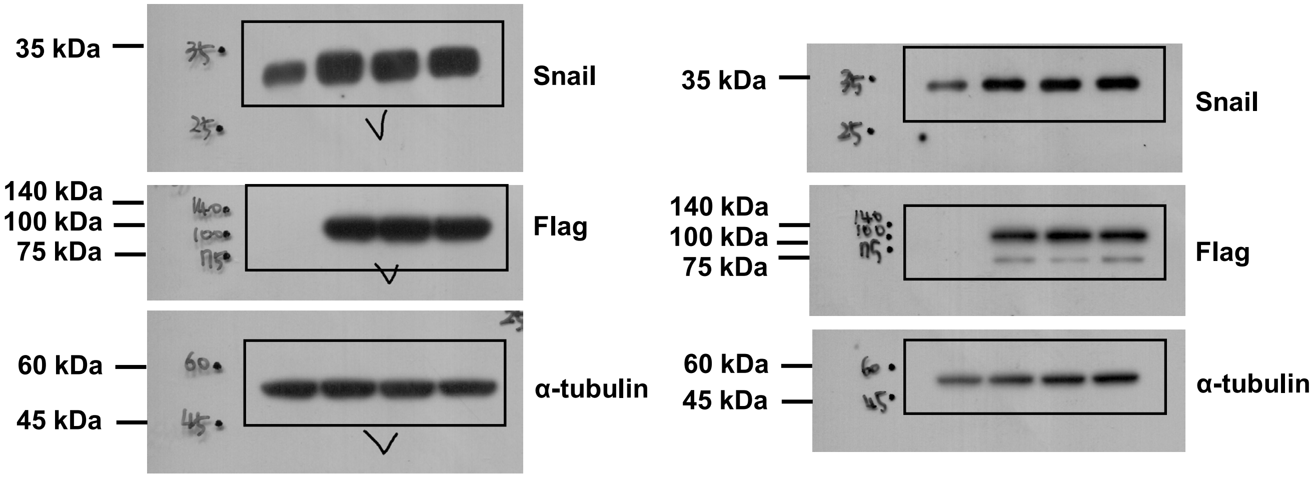

Figure S10

Figure 5D

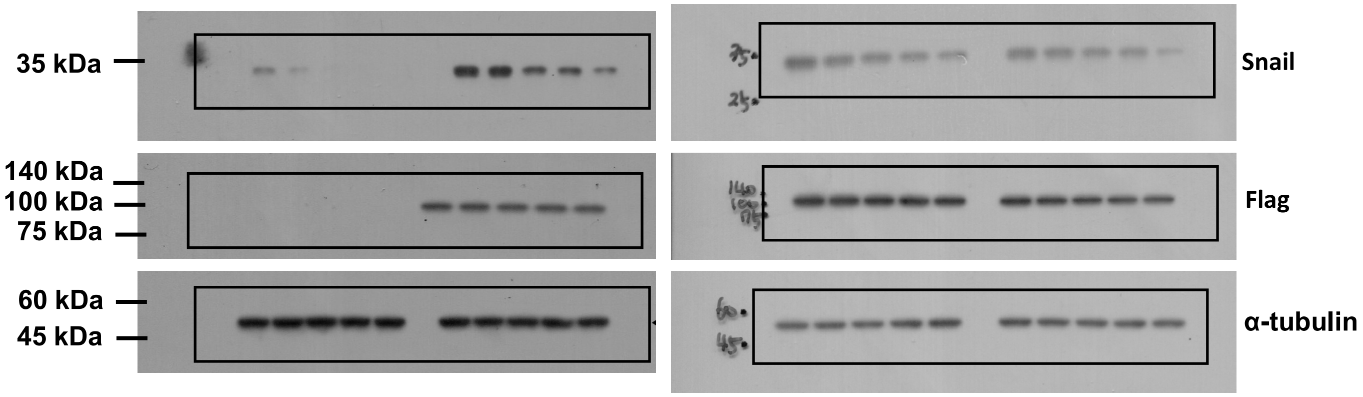

Figure 5E

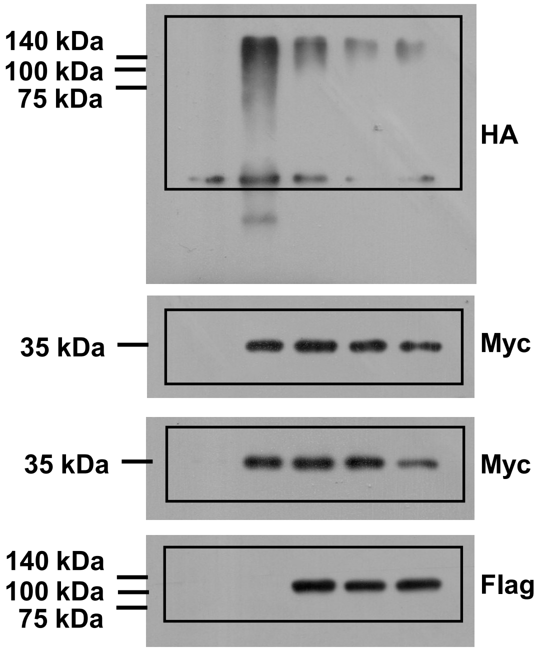

Figure S11

Figure 6A

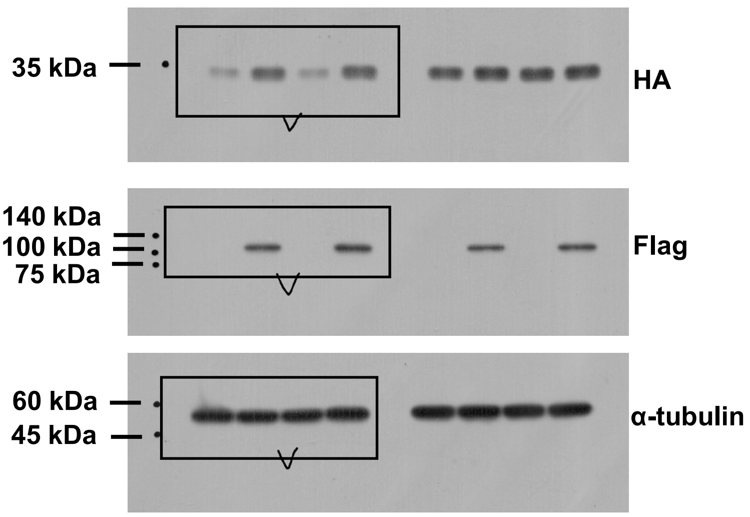

Figure 6B

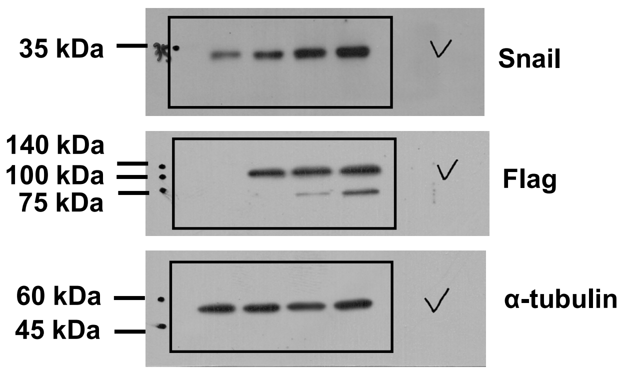

Figure 6C

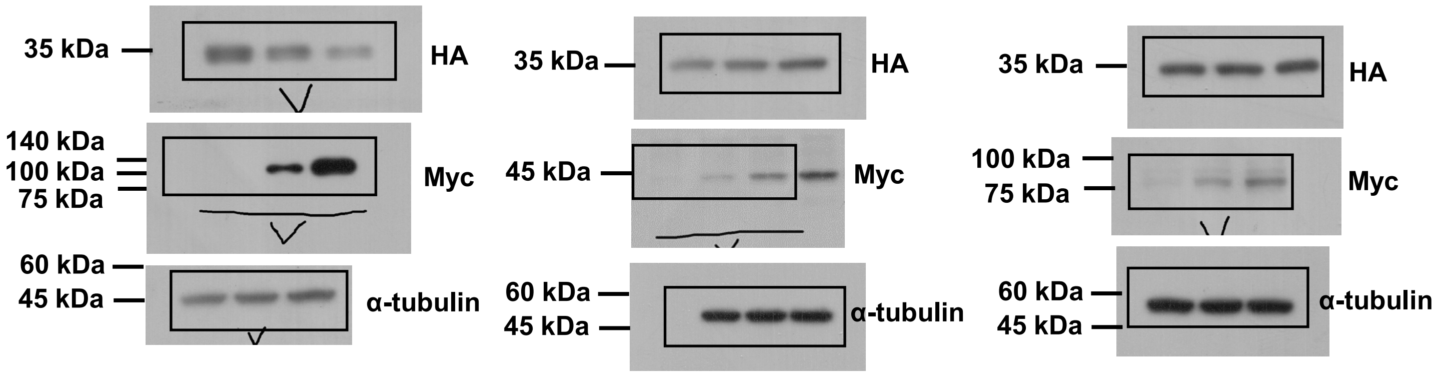

Figure S12

Figure 6D

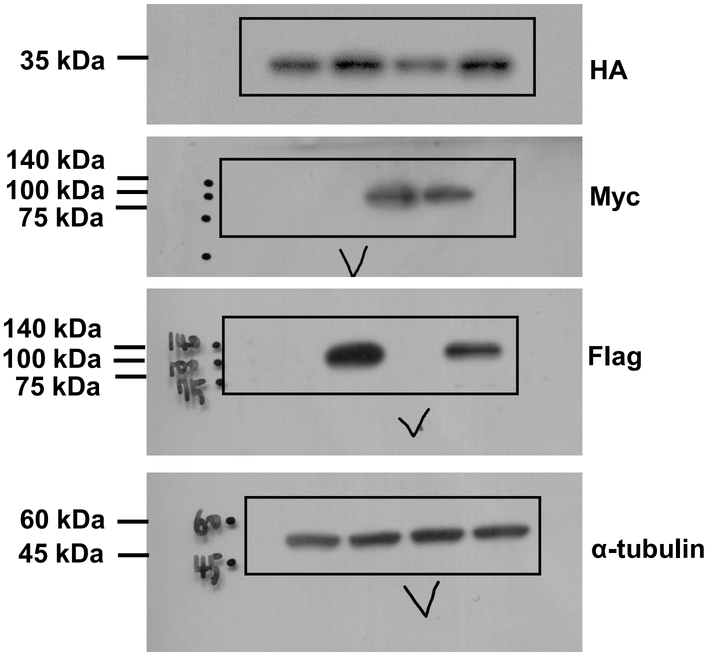

Figure 6E

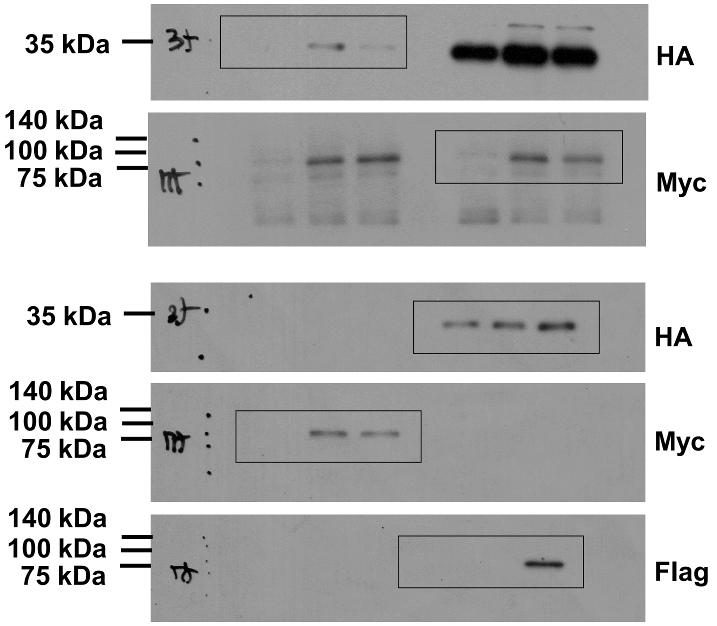

Figure 6F

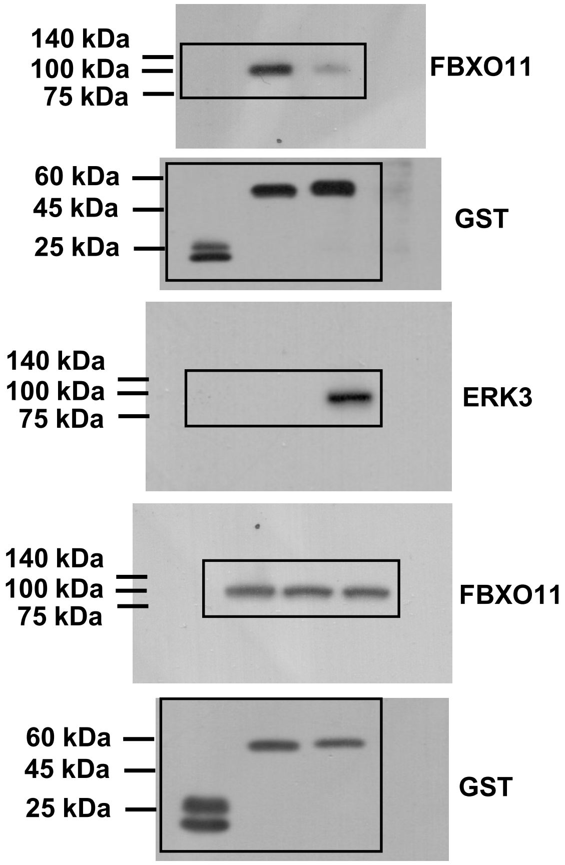

Figure S13

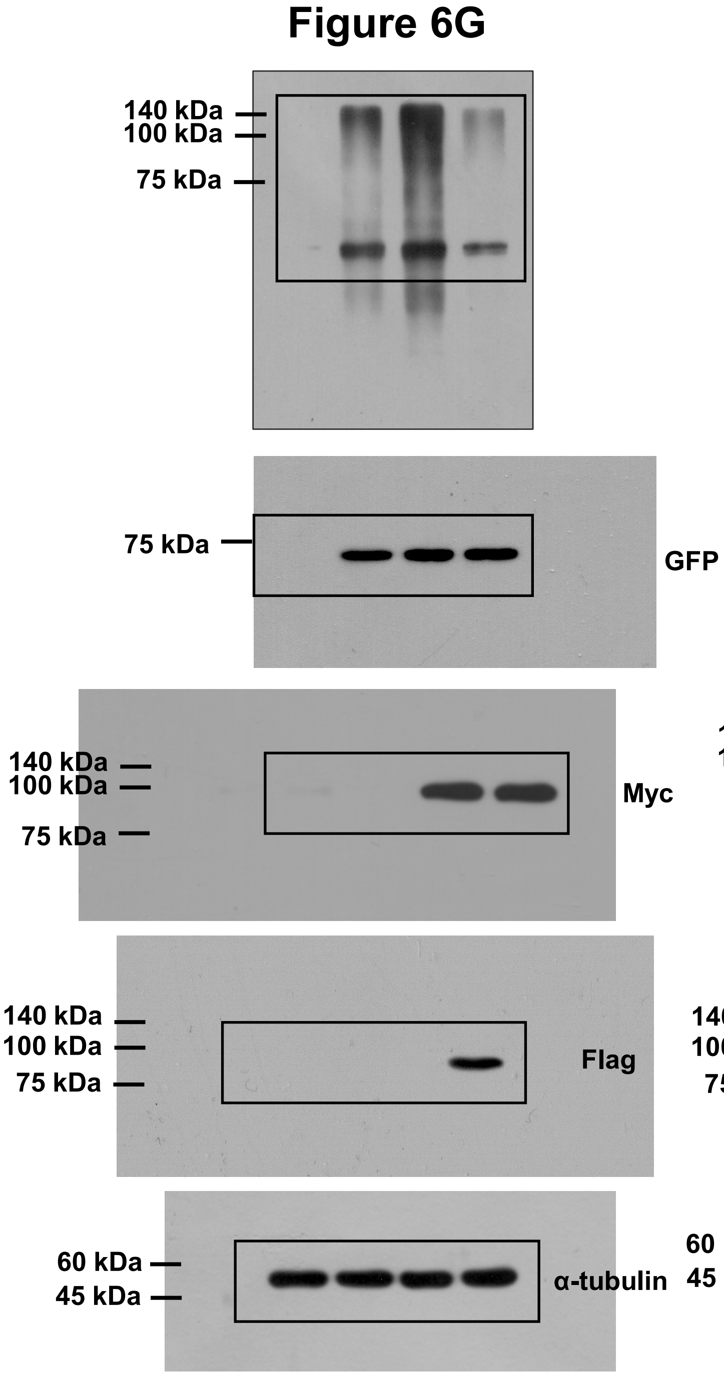
Figure 6G
140 kDa
100 kDa
75 kDa
75 kDa
GFP
140 kDa
100 kDa
75 kDa
Myc
140 kDa
100 kDa
75 kDa
Flag
60 kDa
45 kDa
α-tubulin

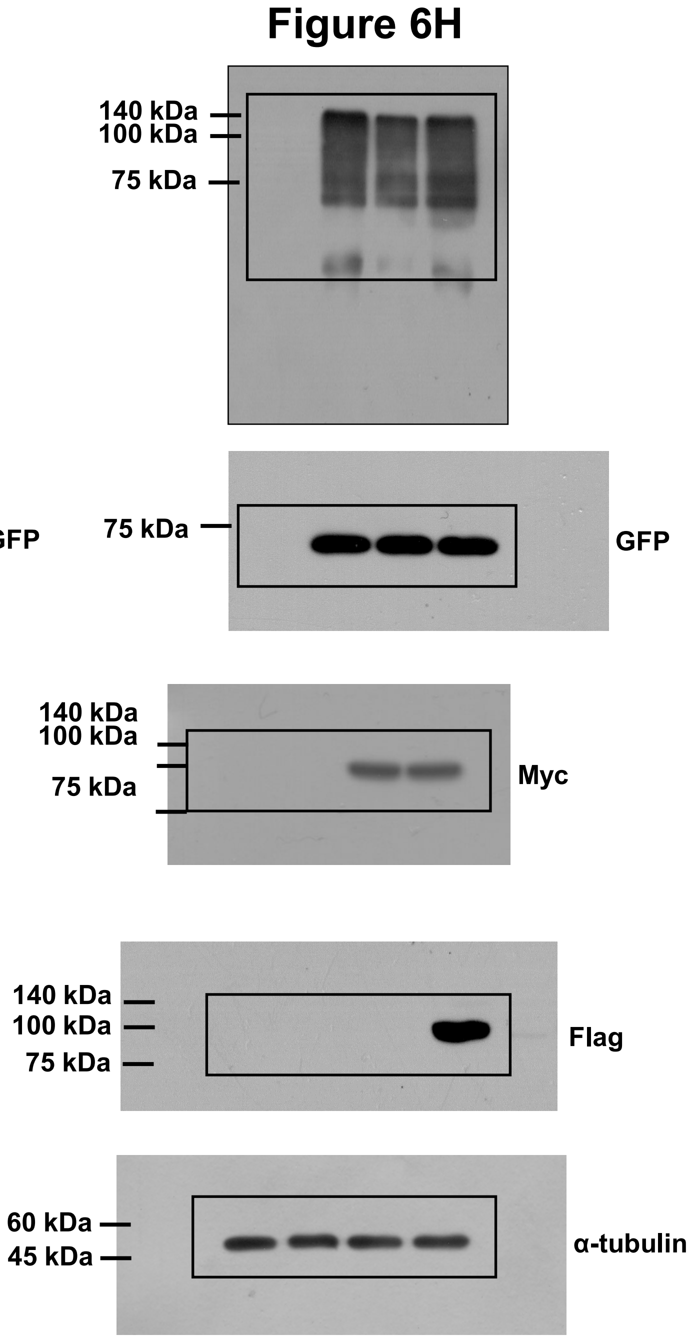
Figure 6H
140 kDa
100 kDa
75 kDa
75 kDa
GFP
140 kDa
100 kDa
75 kDa
Myc
140 kDa
100 kDa
75 kDa
Flag
60 kDa
45 kDa
α-tubulin

Figure S14

Figure 7A

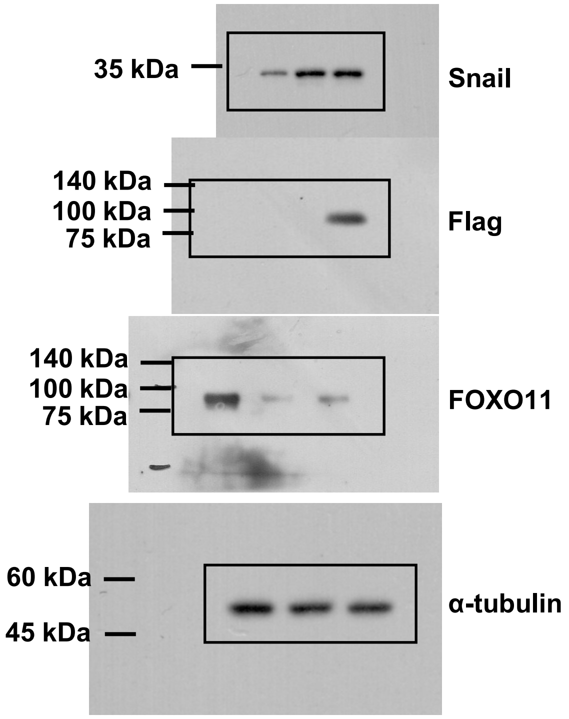

Figure 7B

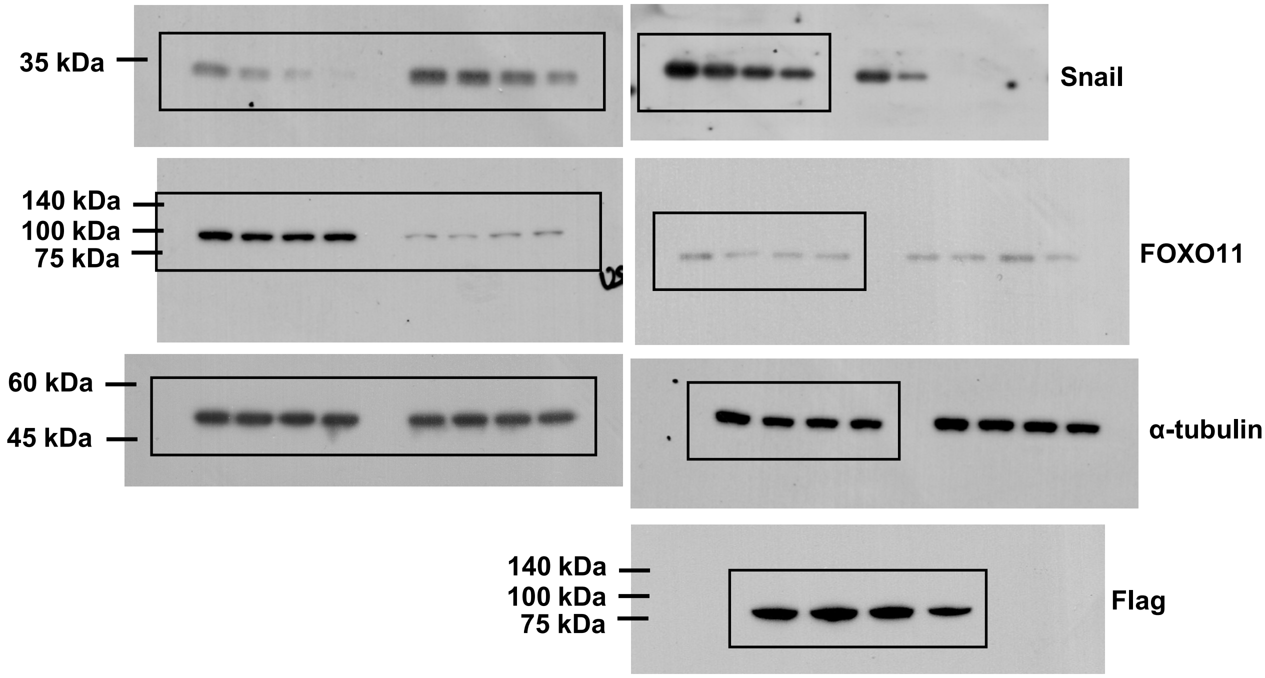

Figure S15

Figure S1A

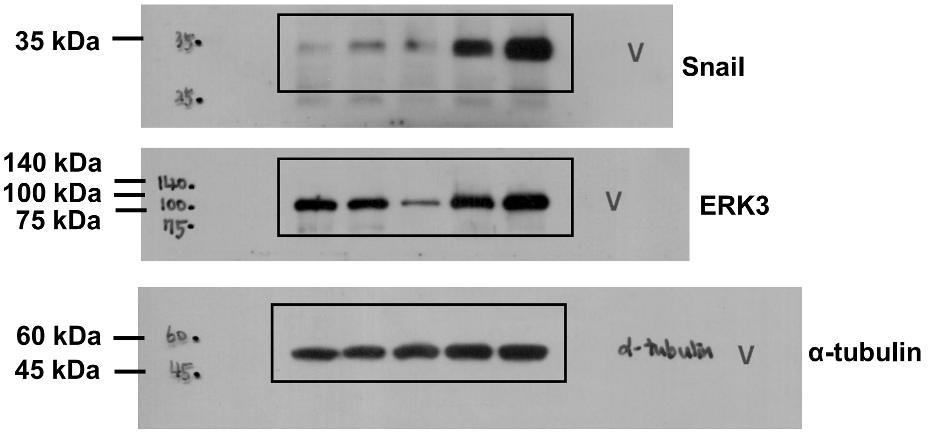

Figure S1B

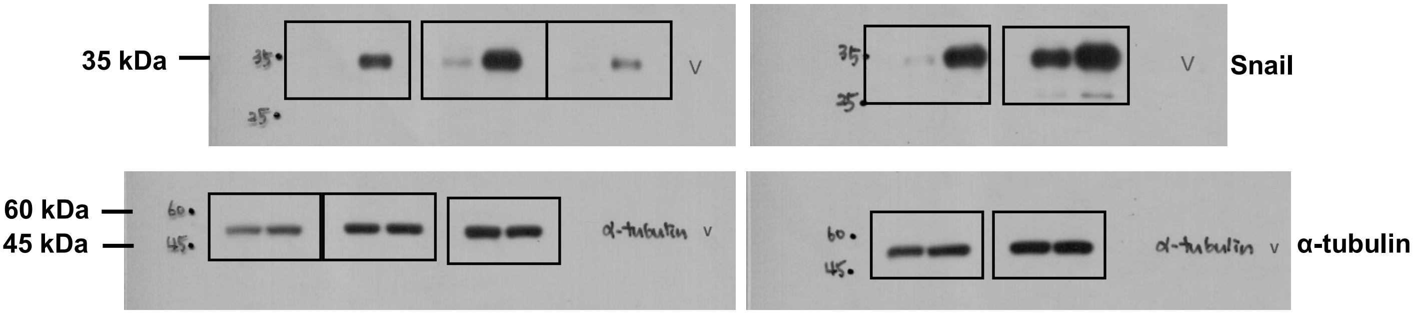

Figure S16

Figure S3B

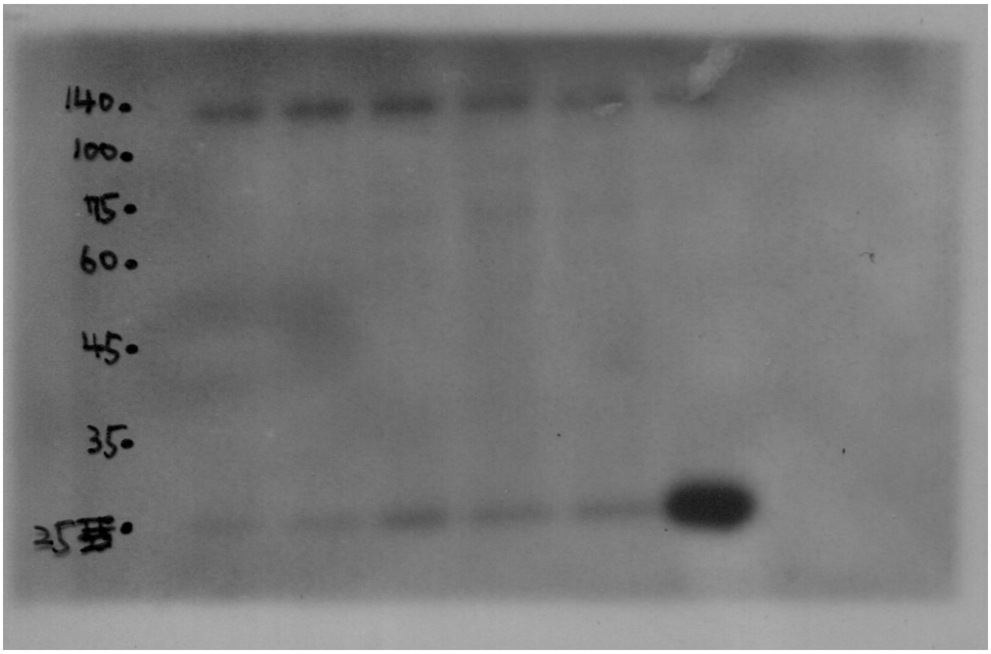

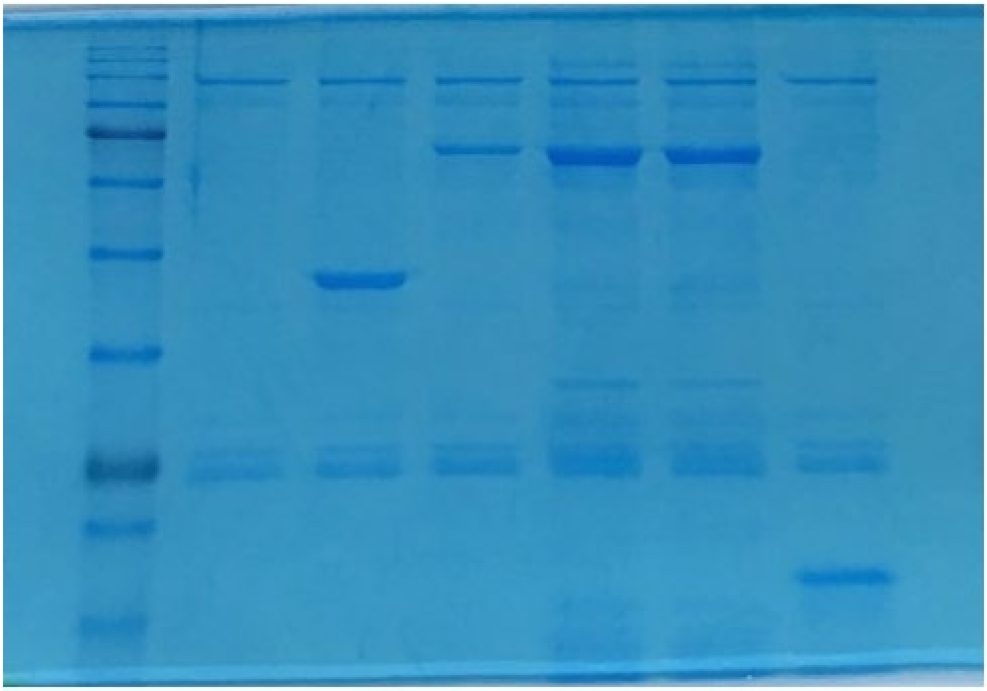

Supplement: Supplementary file 1 [file cancers-16-00105-s001.zip › cancers-2761822-supplementary.pdf]
